# A Review of Woven Tracheal Stents: Materials, Structures, and Application

**DOI:** 10.3390/jfb13030096

**Published:** 2022-07-16

**Authors:** Chen Xu, Yanxue Ma, Haihua Huang, Zheng Ruan, Yuling Li

**Affiliations:** 1 College of Textiles, Donghua University, Shanghai 201620, China; 1215005@mail.dhu.edu.cn (C.X.); yxma@dhu.edu.cn (Y.M.); 2 Department of Thoracic Surgery, Shanghai General Hospital, Shanghai Jiaotong University, Shanghai 200080, China; hhh002559@163.com

**Keywords:** weaving technology, trachea defect, artificial trachea stent, trachea defect, regeneration

## Abstract

The repair and reconstruction of tracheal defects is a challenging clinical problem. Due to the wide choice of materials and structures, weaving technology has shown unique advantages in simulating the multilayer structure of the trachea and providing reliable performance. Currently, most woven stent-based stents focus only on the effect of materials on stent performance while ignoring the direct effect of woven process parameters on stent performance, and the advantages of weaving technology in tissue regeneration have not been fully exploited. Therefore, this review will introduce the effects of stent materials and fabric construction on the performance of tracheal stents, focusing on the effects of weaving process parameters on stent performance. We will summarize the problems faced by woven stents and possible directions of development in the hope of broadening the technical field of artificial trachea preparation.

## 1. Introduction

**Figure 1:**
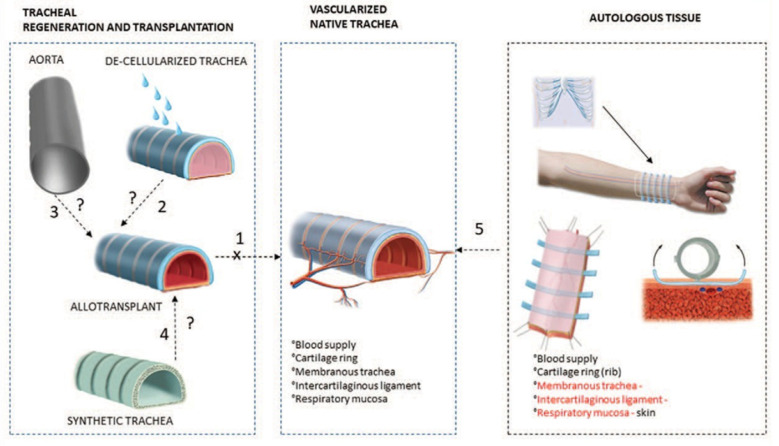
The primary trachea and its alternatives. (1) Trachea allotransplant; (2) Trachea with enzyme-free cells; (3) Aortic allograft; (4) Synthetic tube; (5) Autologous tissue. Figure modified from reference [7], with permission from Wolters Kluwer Health (License Number 501730685), 2020.

Trachea is a pipe connecting throat and external environment, which is essential for swallowing, speaking, and neck movement [1]. Infections, injuries, tumors, and congenital reasons, such as tracheal stenosis after intubation and persistent tracheal fistula, can all lead to issues such as difficulties speaking, mucus discharge, and recurring lung infections [2]. The treatment method for human trachea is determined by the length and size of the defective area [3]. When the length of trachea injury is short, trachea resection in thoracic surgery can be repaired by end-to-end anastomosis [4]. This procedure is no longer appropriate in cases where the damaged trachea measures more than 1/2 the length of the tracheal tissue in adults or 1/3 the length of the tracheal tissue in youngsters [5,6,7,8]. A suitable tracheal substitute is required for successful airway reconstruction. Many ways to develop acceptable tracheal substitutes have been tested in clinical practice over the years, including autologous tracheal transplantation [9,10,11], tracheal allotransplantation [12,13,14,15,16,17,18], and stent implantation [19], as shown in Figure 1. Autologous transplantation is regarded as a tracheal transplantation procedure that causes the least amount of immune rejection and can prevent some potential issues, like infection and cracking [20]. However, using only autogenous tissue makes it challenging to mimic the biomechanical characteristics of a healthy trachea. Lack of donors and immunological rejection are additional issues with allotransplantation. The artificial tracheal stent’s material and structure can be chosen from a variety of options as an appropriate way to get over the lack of grafted tissue. With the optimized tent design and technology, it is possible to create stents that have mechanical, biological, and biological qualities that are comparable to healthy tracheal tissue. Artificial tracheal scaffolds can act as carriers of growth factors and signaling molecules that promote cell development, differentiation, proliferation, and tissue.

Since Montgomery’s invention of the t-tube silicone stent for tracheal stenosis in 1965, a variety of stents have been developed. Freeze drying [21], electrostatic spinning [22], three-dimensional (3D) printing [23,24,25], textile techniques [26,27], and many other methods have been used for the manufacture of tracheal stents. Although these manufacturing methods produce stents with 3D porous structures, they are limited in controlling pore size. The distribution and spatial connection ability often cannot meet the mechanical properties and structural properties of similar natural gas pipelines. As a kind of textile technology, weaving technology composed of intersecting warp and weft yarns has an excellent performance in precise control of pore size, shape, and mechanical properties. By adjusting the process parameters, the woven tracheal stent can be combined into different patterns and shapes, showing unique advantages in simulating the tracheal hierarchy, anisotropy, and mechanical properties. Table 1 summarizes the research advances in weaving technology in the artificial trachea. There are several excellent reviews on the application of textile stents in different organ areas [28,29]. Because of unfamiliarity with the characteristics of woven technology, most researchers have not thoroughly discussed the application of woven technology in artificial tracheal stents. The feasibility of preparing tracheal stents with woven technology has been overlooked in some cases. This review mainly focuses on the application of woven technology in artificial tracheal stent and summarizes the influence of stent materials and woven process parameters on stent performance. We summarize the challenges and future prospects of tracheal braiding, which can provide reference for the design of high-performance stent and help to broaden the application field of artificial tracheal stent.

## 2. Materials Used in Woven Tracheal Stent

Materials are a fundamental component of medical stents. The performance of the stent material has the most direct impact on the ultimate physicochemical function of the stent. The tracheal stent’s key functions are to offer a suitable three-dimensional environment for cell proliferation, effective mechanical support for the trachea’s regenerative, and finally to fulfill the therapeutic objective of widening the constricted trachea or closing the damaged tracheal fistula. The majority of commercial tracheal stents are comprised of silicone or metal, and they offer sufficient mechanical support. However, because these stents are non-degradable, patients need to undergo a second stent grafting procedure. Late issues are more likely when an artificial stent remains in the trachea for a long time (such as granulation, infection). Table 2 summarizes the categories of artificial tracheal stents and compares them. The choice of stent materials has expanded from common inert materials to biomaterials with specific biocompatibility as the materials discipline has advanced. Stent raw material composition is also not limited to a single material; composite materials are widely used in the study of tracheal stents. In addition, prevascularization and tissue engineering strategies loaded with growth factors also eliminate the need to consider only material biological characteristics when selecting scaffold materials. The following factors influence the selection of stent materials:

- Excellent biocompatibility.
- Excellent mechanical properties.
- The degradation rate of the material matched that of the new tissue.
- Immune rejection is within acceptable range.

### 2.1. Non-Degradable Material

**Figure 2:**
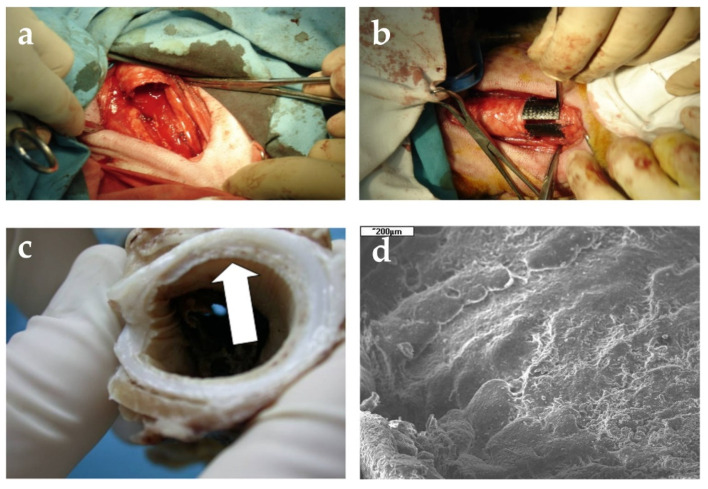
(**a**) Resection of tracheal segment consisting of four rings; (**b**) Reconstruction of tracheal defect with cylindrical implant; (**c**) Tissues covering outer surface of the implant at the anastomosis site; (**d**) Tissues covering inner surface of the implant. Figure modified from reference [8], with permission from Wojciech Ścierski, Copyright © 2018 Wojciech Ścierski.

Due to the limitation of material selection, the earliest stents were made of non-degradable silicone material [42,43]. The majority of tracheal stents currently used in clinical practice are made of silicone and metal, such as Dumon (Novatech, France), Ultraflex (Boston Scientific), and Palmaz (Johnson and Johnson). The silicone T-tubes used in surgical procedures are lightweight and flexible, with little stent displacement. Commercial Dumon stents are widely used, but their stent fixation is dependent on pressure and frictional resistance between tracheal wall and stent tubing nail, and the procedure is more difficult. Chen et al. [44] calculated the efficacy and complication rate of implantation of a silicone Dumont stent, and came to the conclusion that Dumon stent had a complication rate of about 20%. Metals are also commonly used in implant medicine. Metal stents have excellent natural mechanical properties and can effectively support human organs. Biotextiles have developed rapidly in the last two decades. The combination of polymer materials and biomedical engineering provides the necessary conditions for the design and development of an ideal artificial trachea. Wojciech Ucierski et al. [8] prepared cylindrical tracheal implants from carbon fibers and observed the effect of tracheal reconstruction in sheep, as shown in Figure 2. 

### 2.2. Biodegradable Synthetic Materials

Biodegradable polymer materials are excellent candidates for the manufacture of tracheal stents. The advantage of biodegradable polymers is that they provide a transient mechanical support for the damaged organ, but the material of the stent can degrade or reabsorb over time, avoiding secondary surgery. The degradation of these polymers is mainly caused by chemical bond hydrolysis. The rate of stent degradation is influenced by factors such as pH and infection in the surrounding environment, and the scaffold degrades at inconsistent times in different tissue environments. The accumulation of acidic degradation products of these synthetic polymeric materials leads to a decrease in the pH of the tissue microenvironment [45], which in turn triggers inflammation. Therefore, the surface of the stent can be coated with drugs to reduce the risk of complications such as rejection reactions and infections after stent implantation. A major challenge in using polymers as organ stent materials is the lack of binding sites on the surface of these fibers, which prevents them from bonding to cells. Researchers have attempted to adjust the surface properties of stents using methods such as surface modification or covering the fiber surface with a cytoplasmic matrix material to promote cell adhesion growth and tissue regeneration. The materials applied to woven tracheal stents are PPDO, PDO, and PLA.

#### 2.2.1. PDO

PDO is a well-studied material. Some sutures and surgical stents made from PDO have been proved safe and effective by Food and Drug Administration (FDA) [46]. Ether bond in its molecular chain has good elasticity and mechanical toughness, high polymer regularity, high elongation, and good toughness of fiber. PDO can be used as a raw material for medical textiles such as surgical sutures, orthopedics and prostheses, stents, and tubes [47]. The proper degradation rate of PDO is well coordinated with the healing process of tracheal tissues, and the stent provides good mechanical support in the initial stage of placement, while it is gradually degraded until complete tissue recovery as the tissue cells grow in adhesion. The percentage of the crystallization phase of the PDO has been increasing since the degradation begins, and it is accompanied by the decline of brittle rise and toughness of materials [48]. This is because the convergence of the crystal is increasing, the molecular chain is more closely organized, the interaction between the molecules increases, and the chain movement becomes difficult. 

Heat treatment has been proven to affect the crystallization and mechanical properties of the material, and the low temperature annealing helps to make up the mechanical energy of the PDO material [49]. This may better match the degradation of the implant and the gradual loss of mechanical energy, thereby making a variety of regeneration strategies in a single biological material system.

#### 2.2.2. PPDO

PPDO is usually formed by ring-opening and heated polymerization of PDO in the presence of organic catalysts [50]. Due to the presence of ether bonds on the macromolecular chain, PPDO has good mechanical strength and flexibility. PDO has good macromolecular regularity, high flexibility, and high orientation and crystallinity after spinning into monofilaments, so it has good tensile breaking properties and can overcome the twisting, stretching, and frictional effects during the weaving process [45]. As an emerging biodegradable material, PPDO has good biocompatibility. The degradation products of PPDO are less acidic than PLA [51], and its final degradation products are water and carbon dioxide. The complete degradation time of PPDO is 6 months, which matches well with the healing and regeneration cycle of tracheal tissue. In the process of degradation, the crystallinity of PPDO increased first and then decreased, and its crystal structure was not significantly changed. However, the high crystal degree and the low melt strength of PPDO are restricted to its application for drug delivery. To improve the thermal stability and mechanical properties of PPDO, various modi-fi-cation methods such as copolymerization [52] and blending with other polymers have been tried. 

#### 2.2.3. PLA

One of the most widely used biodegradable polymer materials is polylactic acid and its derivatives, copolymers, which can be made by fermenting sugars obtained from renewable sources, such as sugar cane or corn starch, and is therefore more effective and less toxic when used in humans. PLA comes in several forms, and its properties can be modulated by changing the chemical composition of the polymer [53,54,55]. For example, Poly L-lactic acid (PLLA) is semi-crystalline, and its vitrification temperature is 65 °C and melting temperature is about 175 °C. Poly(D, L-L actide) (PDLLA) was amorphous and its vitrification temperature ranged from 55 °C to 60 °C. PLLA is more biocompatible because it is a natural isomer [56]. Poly (lactic acid) has good mechanical properties (high tensile strength and rigidity), but also has some disadvantages (low elongation at break, low toughness) which limit its application under high stress requiring plastic deformation. 

The degradation of PLA acid depends on many factors, such as molecular weight, pH, crystallinity, and microorganism [57]. PLA has a relatively long hydrolysis period due to spatial effects, in which the alkyl group prevents water from attacking the polymer. PLA stents or fibers do not begin to degrade until about 12 months after exposure to in vivo conditions, and do not fully degrade until about two years [58]. During the body’s degradation process, PLA material can be broken down into water-soluble polymers or small fragments that can be excreted directly from the body. Therefore, PLA stents may not be completely degraded to carbon dioxide and water in the human body. At present, the application of PLA in tracheal stents is mainly due to its reliable mechanical properties rather than due to its degradability. Because the hydrolysis of the PLA copolymer is unstable, the stent can be fully absorbed within a pre-designed time frame (from a few months to a few years) by modifying its physicochemical structure [59]. In most cases, PLA is not the only material constituting stents, but is used as a coating for stents or copolymerized with other rapidly degraded monomers (such as PGA) [60]. Studies in rabbits have shown that PLA can achieve effective sarcomere inhibition when used as a coating [61].

### 2.3. Biodegradable Natural Materials

Compared with synthetic materials, natural biological materials such as protein and polysaccharide-based materials have better biocompatibility. The properties of these natural materials are more suited to be biologically compatible with tracheal tissue than to provide adequate mechanical support [62]. For example, gelatin materials have biological properties similar to those of natural tissue cytoplasmic matrices, but their poor mechanical properties limit their application. Therefore, gelatin may not be used as a stand-alone stent material in some studies, but rather as a coating or additive to synthetic polymers or metal stents that can improve the biocompatibility of the stent while maintaining its established physical properties [63]. Bacterial cellulose can also be considered as one of the alternative materials for tracheal stents because of its high flexibility and tensile strength (Young modulus of 15–18 GPa) [64]. Although the mechanical properties of bacterial cellulose are very similar to those of rigid and flexible tracheal stents, unfortunately, bacterial cellulose has not yet been used to prepare tracheal stents.

Currently, a wide range of materials is available for artificial tracheal stents. However, probably due to unfamiliarity with the woven technology, most researchers prefer other manufacturing techniques when choosing the preparation process of stents. It is undeniable that, as one of the oldest techniques, the woven technology has advantages in building structural anisotropy and in precise control of size and shape. The design and development of woven tracheal materials have considerable research value.

## 3. Structure of Woven Tracheal Stent

Stent structures play an important role in tissue regeneration. The textile structure composed of polymer fiber has good 3D structure and mechanical properties, which can meet the requirements of porous structure and support the performance of human medical stents, and has suitable viscoelastic characteristics of rigidity and flexibility. The ideal stent needs to have mechanical properties similar to those of flexible human tissue, as well as adequate radial support. In addition, the stent needs to be placed in a sheath and then released into the body. During the compression of the tracheal stent, it is important to avoid severe mechanical property loss of the stent. Stent structural parameters such as three-dimensional shape, thickness, dimensions, total porosity, and pore size distribution affect cell differentiation and proliferation, immune system response, and tracheal tissue regeneration.Therefore, the design and manufacture of stents should be carried out according to the needs of the patient [65]. 

**Figure 3:**
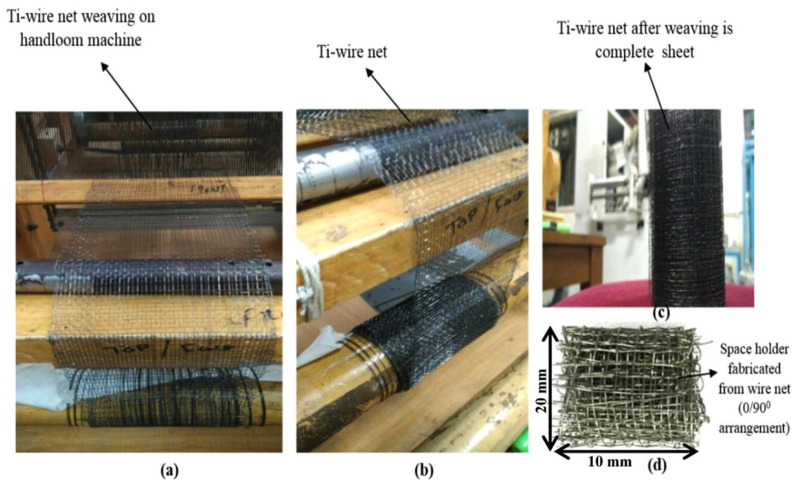
Ti-wire sheet fabrication from handloom machine: (**a**) weaving of Ti-wire net over handloom; (**b**) side image of (**a**); (**c**) Image after titanium mesh weaving; (**d**) space bracket after titanium mesh weaving. Figure modified from reference [67], with permission from Elsevier (License Number 5337841389847), 2019.

Weaving technology has a history of thousands of years and has experienced the stages of primitive weaving, ordinary loom, and automatic loom. There are many types of weaving machines, such as shuttle looms, rigid rapier looms, and air-jet looms. The diversity and selectivity of the woven structures allow us to control the microstructure and cellular distribution of the final scaffold by regulating the pore size and spatial distribution of interconnections, such as controlling fiber size and orientation, pore size and geometry, pore interconnections, total porosity, and surface topography [66]. Figure 3 illustrates a Ti-wire sheet fabrication from a handloom machine.

## 3.1. 2D Weaving and 3D Weaving

**Figure 4:**
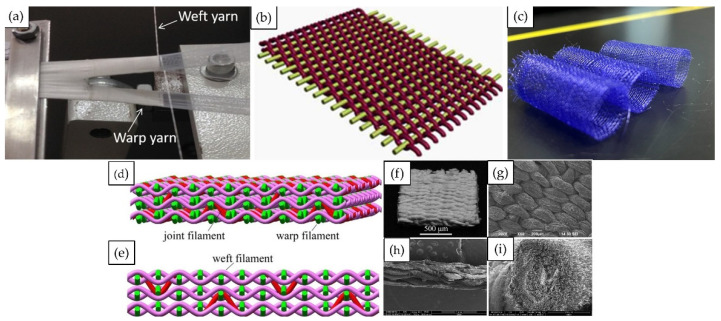
The techniques used for fabricating traditional 2D (**a**–**c**) and 3D l (**d**–**f**) waving stent: (**a**) Weaving prototyping system; Figure modified from reference [37], with permission from Elsevier (License Number 5338130678249), 2019. (**b**) Schematic diagram of the 2D woven stent. Adapted with permission from [69]. Copyright 2017 American Chemical Society. (**c**) 2D woven tubular stent. (**d**,**e**) Schematic diagram of the 3D woven stent. (**f**–**i**) SEM image of 3D stent. (**d**–**i**) modified from reference, with permission from Elsevier [70] (License Number 5338120962811), 2016.

The hollow structure of tubular supports can be realized in two-dimensional weaving. Two-dimensional looms can not only create flat fabrics similar to cloth, but can also be used to weave three-dimensional hollow tubular fabrics. This three-dimensional fabric is mainly woven using the flattening-weave-reduction method. The fabric consists of one set of warp yarns and two sets of weft yarns which act as both surface weft yarns and lining weft yarns to form the upper layer of the fabric. The bottom layer of the fabric is made up of warp and weft yarns, which are repeatedly interwoven between the two surface layers [68]. This seamless, woven 3D Tubular support can be completed directly on the weaving equipment without the need for advanced manual skills and experience. The one-piece woven weave avoids the possible adverse effects of woven fabric selvedge. Figure 4 shows two dimensional looms, three dimensional looms, and the scaffolds made from them.

### 3.2. Woven Patterns

**Figure 5:**
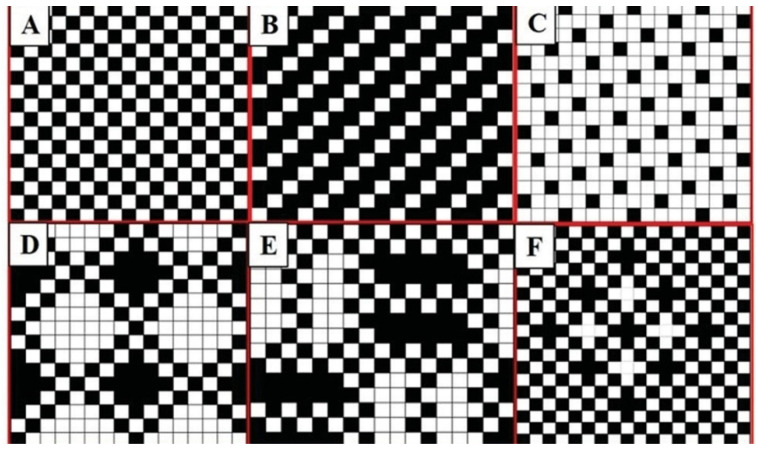
Different kinds of woven structures. (**A**) plain weave, (**B**) twill weave, (**C**) satin weave, (**D**) honeycomb weave, € fancy cord weave, and (**F**) fancy mesh. The black point means that the warp yarn is over the weft yarn, and the white point means that the weft yarn is over the warp yarn. Figure modified from reference [71], with permission from Elsevier (License Number 1242110-1), 2020.

Various woven patterns and their corresponding properties show the potential to mimic the specific properties of human tissues. Woven patterns also influence cell proliferation and differentiation, as cells are adsorbed to the surface of the scaffold material for proliferation and differentiation. There are many types of woven structures, and Figure 5 shows some basic woven structures as well as complex woven patterns.

Common woven patterns are plain, twill, satin, and so on. Plain fabrics are made up of two different sets of yarns, usually interwoven. The longitudinal yarns are called warp yarns, while the weft passes through them in a transverse direction [66]. The yarn at the interlace is slightly deformed, forming a curved wave shape. Under the action of an external force, the bending wave is straightened and the yarn is stretched. However, the deformation of bending wave is small, so the radial tension of braided support is large. Ordinary woven tubular fabric is plain weave and twill weave structure, adjacent weft yarn has approximately parallel distribution. When supported by radial pressure, warp yarn and weft yarn are independent of each other along the stress direction, and the yarn is approximately a straight line. Bending stiffness is a small, easy to compress deformation, and its radial support force is difficult to meet the requirements of the trachea support.

In order to solve the above problems, some novel patterns, such as fancy leno structures and duplex weft structures, were used for the preparation of holders. Li et al. [72] designed a double-layer woven tubular fabric by changing the structure of the ordinary woven fabric, and enhanced the radial support force of the support by adding a layer of weft monofilament floating on the outer layer of the artificial air tube wall. However, because the aperture of ordinary tubular woven fabric is too small, it is difficult to meet the growth of cell group attachment, and the warp and weft yarn of woven fabric prepared by plain weave and other tissues are arranged in parallel, which is easy to slip when extruded by external forces, so the structure of the support is not stable. Fu et al. [73] designed a kind of change in the structure of the gauze woven tracheal stents, based on the original yarn organization ground every transform position into two warp, and the wringer position between the adjacent two ground group stagger, gauze organization unique bending increases the bending stiffness, adjacent warp when a force can resist external force, together in stent and porosity. Under the premise of aperture size, the overall structure is stable and not easy to slip, and the radial support force and elastic recovery capacity of the tracheal stent areas is significantly improved. 

### 3.3. Weaving Process Parameters

The weaving technology process parameters are closely related to the mechanical properties and tissue repair effect of the stent and affect all aspects of the stent performance. Some process parameters, such as material diameter, density of warp, and weft yarns, affect the porosity and pore size of the fabric. For example, the decrease of fiber diameter is beneficial to increase the specific surface area of the scaffold, which increases the proteins and biological factors adsorbed by the scaffold and facilitates the cell proliferation. 

An important factor affecting fabric looseness and porosity is the count of warp and weft yarns per square inch. If the warp and weft yarn density of the woven fabric is low, this means that there are more pores to support cell attachment and growth, but the lower warp and weft yarn density leads to a looser support structure, resulting in insufficient friction between the warp and weft yarns, which eventually leads to warp and weft yarn slippage and unstable support structure. If the support is too small, it will easily shift after implantation, and if the support is too large, it will cause compression on the trachea wall due to excessive radial force. Therefore, the basic process parameters should be determined in advance when weaving the woven stent. The weaving process parameters can be adjusted to match the native tissue in morphological scale and mechanical characteristics.

## 4. Challenges and Future Prospects

Weaving technology has a broad application prospect in the field of air duct engineering. Through the design of the woven process structure, the precise control of pore and mechanical properties is realized. At present, the overall level of woven tracheal stents is still in the preliminary laboratory development stage. Like other weaving processes, stents prepared by the woven process need to take into account the vascularization, epithelialization, antibacterial, mechanical properties, and the size of the stent. Although woven tracheal stents have their structural advantages, the feasibility of stents for human tracheal graft regeneration still needs to be evaluated from several perspectives.

The weaving process parameters have a great influence on the mechanical properties of the stent after implantation. Currently, the woven tracheal stent textile pattern and layer structure are relatively simple, and more fabric structures need to be considered to simulate the mechanical properties of native organs and tissues, thus increasing the degree to which the stent matches the mechanical properties of other surrounding tissues. If the rigidity of the tissue substitute is not designed properly, it may cause a foreign body reaction, leading to pain or inflammation in the patient. This challenge can be addressed by research into new degradable materials. If the weaving process is complex, a combination of experimental testing and finite element simulation can also be used to explore the relationship between different structures of the stent and the mechanical properties of the stent, and to optimize the structural design and preparation of the stent based on the fitting results of experimental testing and simulation [74].

The regulation of cell behavior and tissue regeneration by weaving technology is limited. Existing improvement methods combine woven technology with other weaving techniques, such as electrostatic spinning and 3D printing. Since the trachea is directly connected to the outside world, the risk of infection at the anastomosis between the implant and the remaining airway is high. The woven technology can be combined with growth factors and drugs to achieve further optimization of the stent. Drug-eluting [75], surface modification [76,77], coating treatment [78,79], and tissue engineering in vitro culture [80,81] are all conducive to the vascularization and improvement of antibacterial performance of stents.

The feasibility of the application of woven support is also related to the progress of textile machinery. Woven products used in the industry are mass-produced. However, most of the medical scaffolds based on woven technology are made by hand, which cannot be automated and efficiently produced. Therefore, the improvement of woven instruments will be beneficial to the production and application of woven stents.

## 5. Conclusions

This review consists of three parts. The first part shows the materials used in woven tracheal stents and their characteristics. The second part discusses which factors of the weaving process can affect the performance of artificial tracheal stents. In addition to this, we present some of the current challenges facing woven stents and possible future directions.

The preparation of stents with biomechanical properties is crucial for the repair and regeneration of the trachea. Woven tracheal stents are of research interest in the field of artificial tracheal stents because of their advantages of forming a three-dimensional porous structure and stable mechanical properties. The material of the stent has a great influence on the final performance of the stent and therefore needs to be selected according to the actual tracheal needs. In the design and preparation of stents, it is hoped that the special features of tracheal stents can be achieved by regulating the weaving process. For the structure of the fabric, there are many complex tissues that are not cited. It is expected that future work can focus more on this area.

Currently, research on woven stents is focused on electrostatic spinning and 3D printing. There are few studies on the preparation of artificial tracheal stents by woven technology. Therefore, it is necessary to gradually improve the performance evaluation system of woven tracheal stents and explore the relationship between woven process parameters and stent performance so that the weaving technology can better play its role in the process of tracheal stent preparation. The development of woven tracheal stents involves multidisciplinary fields such as biology, materials science, and textile science. We believe that with the optimization of biomaterials and improvement of the weaving process, combined with other weaving processes, the research of artificial trachea will be greatly developed, thus bringing new hope to the majority of patients with tracheal diseases.

## Figures and Tables

**Table 1:** Research advances in woven technology in artificial stent.

Year	Material	Application Areas	Research Contents	Reference
2013	Alginate/polyacrylamide/poly(ε-caprolacto/ne)	Articular cartilage	Formation of a dual-network “tough-gel” to establish load-bearing and tribological properties similar to native cartilage	[30]
2018	Hydrogel/polyacrylonitrile/N, N-Dimethylformamide/polycaprolactone/polyurethane	Heart valves	Integration of woven fiber networks into bioactive hydrogels to produce stents with anisotropic biomechanics and valve ECM like microenvironment	[31]
2018	Poly-l-lactide/poly-l-lactide-co-E-caprolactone	Bone	The permeability and porosity of the stent were evaluated by adjusting the material combination, weave configuration, and fiber Geometry	[32]
2014	Polyester/nitinol	Endovascular	Abrasion resistance testing of intravascular fabrics and metal stent grafts.	[33]
2021	Hydroxyapatite/polylactic acid/poly(L-lactide-co- ε-caprolactone/silk fibroin	Tendon-to-bone	The combination of electrostatic spinning and weaving technology enables gradient release of calcium ions, stents are structurally anisotropic and promote osteogenic differentiation and osteoblast proliferation in rats	[34]
2021	Bioactive glass/silver nanoparticles	Bone	Woven structures are used to stimulate the growth of cells by virtue of their balanced yarns intersections on the structures’ surfaces. Stents loaded with g bioactive glass containing Ag 0.5% demonstrated remarkable biomineralization.	[35]
2018	Poly (lactic acid)/hydroxyapatite	Bone	The 3D architecture of woven stent supports the differentiation of the hMSCs into osteoblast cells and enhances the production of mineralized bone matrix	[36]
2019	Silk fibroin/heparin	Heparin	The filamentous silk fibroin/heparin stent uses woven bifurcation technology and steam/air treatment to achieve anticoagulant properties and improve permeability.	[37]
2022	α-Mangostin/Polycaprolactone	Cardiac	Woven nanofiber yarn stents exhibit customizable flexible structures, excellent mechanical strength, proper cell adhesion, and degradation properties in vitro.	[38]
2014	Silk	skin	Significantly higher final tensile strength, elongation at break, and suture retention strength of silk woven stents prepared by enhanced lyophilization with degumming	[39]
2018	Poly(caprolactone)- Collagen/Poly-L-lactic Acid/	Vascular	The patterned woven structure promotes protein adsorption, as well as cell attachment and spreading	[40]
2020	poly-(L-lactic acid)/polypyrrole/copper/platinum	nerve conduits	The design of the braided stent can be used to incorporate conductive materials into polymer yarns to develop electrically stimulable nerve conduits	[41]

**Table 2:** Summary of different fibers used in artificial tracheal and their advantages and disadvantages.

Type of Material		Fibrous Material	Advantages	Disadvantages
Non-biodegradable		Silicone Nitinol	High mechanical strength Not suitable for children or patients with benign diseases	Difficulty of stent removal The possibility of restenosis
Biodegradable	Synthetic	PLA PDO PCL PPDO	Moderate mechanical strength and toughness Non-toxicity of degradation products	Poor cell affinity Differences in the degradation rate of different materials
	natural	Gelatin Silk fibroin	Biocompatibility Minor immune rejection of a material by the body	Low mechanical strength Uncontrolled rate of degradation

Abbreviations: PLA: polylactic acid, PDO: polydioxanone, PCL: polycaprolactone, PPDO: poly (p-dioxanone).

## Data Availability

Not applicable.
